# Crimean-Congo Hemorrhagic Fever Knowledge, Attitudes, Practices, Risk Factors, and Seroprevalence in Rural Georgian Villages with Known Transmission in 2014

**DOI:** 10.1371/journal.pone.0158049

**Published:** 2016-06-23

**Authors:** Ashley L. Greiner, Nana Mamuchishvili, Natia Kakutia, Kendra Stauffer, Marika Geleishvili, Nazibrola Chitadze, Tamar Chikviladze, Khatuna Zakhashvili, Juliette Morgan, Stephanie J. Salyer

**Affiliations:** 1 Epidemic Intelligence Service, United States Centers for Disease Control and Prevention, Atlanta, Georgia, United States of America; 2 Division of Global Health Protection, Center for Global Health, United States Centers for Disease Control and Prevention, Atlanta, Georgia, United States of America; 3 National Center for Disease Control and Public Health, Tbilisi, Georgia; 4 South Caucasus Country Office, Center for Global Health, United States Centers for Disease Control and Prevention, Tbilisi, Georgia; 5 R. G. Lugar Center for Public Health Research, Tbilisi, Georgia; University of Minnesota, UNITED STATES

## Abstract

In 2014 the highest annual case count of Crimean-Congo hemorrhagic fever (CCHF) was detected in Georgia since surveillance began in 2009. CCHF is a high-fatality hemorrhagic syndrome transmitted by infected ticks and animal blood. In response to this immediate public health threat, we assessed CCHF risk factors, seroprevalence, and CCHF-related knowledge, attitudes, and practices in the 12 rural villages reporting a 2014 CCHF case, to inform CCHF prevention and control measures. Households were randomly selected for interviewing and serum sample collection. Data were weighted by non-response and gender; percentages reflect weighting. Among 618 respondents, median age was 54.8 years (IQR: 26.5, range: 18.6–101.4); 215 (48.8%) were male. Most (91.5%) participants reported ≥1 CCHF high-risk activity. Of 389 participants with tick exposure, 286 (46.7%) participants handled ticks bare-handed; 65/216 (29.7%) knew the risk. Of 605 respondents, 355 (57.9%) reported animal blood exposure; 32/281 (12.7%) knew the risk. Of 612 responding, 184 (28.8%) knew protective measures against CCHF and tick exposures, but only 54.3% employed the measures. Of 435 serum samples collected, 12 were anti-CCHF IgG positive, indicating a weighted 3.0% seroprevalence. Most (66.7%) seropositive subjects reported tick exposure. In these villages, CCHF risk factors are prevalent, while CCHF-related knowledge and preventive practices are limited; these findings are critical to informing public health interventions to effectively control and prevent ongoing CCHF transmission. Additionally, CCHF seroprevalence is higher than previously detected (0.03%), highlighting the importance of this disease in the South Caucuses and in supporting ongoing regional investigations.

## Introduction

Crimean-Congo hemorrhagic fever (CCHF) is a zoonotic, viral disease of the *Bunyaviridae* family, primarily transmitted by the *Hyalomma* tick [1]. Transmission occurs from the bite of an infected tick or from crushing an infected tick with bare skin. Secondary transmission has been reported from contact with infected animal blood or tissues, or by ingesting unpasteurized milk. Human-to-human transmission can occur from exposure to infected blood or bodily fluids; however, this is typically reported in healthcare settings [2].

Although animals and ticks do not exhibit clinical signs of CCHF infection, about one out of five humans infected with the virus develop clinically overt illness [3]. In humans the disease presents as a non-specific febrile illness that can rapidly progress into a hemorrhagic syndrome, leading to multi-organ failure and death in severe cases. The reported case fatality rate has varied from 5% to 60% [1, 2, 4–7]. CCHF’s clinical severity, transmissibility, and infectiousness are responsible for its categorization by the National Institute of Allergy and Infectious Diseases as a Biodefense Category A pathogen, the highest risk to national security and public health [8].

Located in the South Caucuses, the country of Georgia is surrounded by neighboring countries where CCHF transmission is endemic [4, 9, 10]. Almost half the population of Georgia resides in rural regions and is employed in agrarian activities, which may put them at risk for CCHF [11]. CCHF surveillance started in Georgia in 2009, when the disease reporting tool, the Electronic Integrated Disease Surveillance System (EIDSS), was established nationally. EIDSS is used as part of the national surveillance system to report notifiable diseases; CCHF reporting occurs when physicians at Georgian healthcare facilities suspect a patient of having CCHF and report this through EIDSS, which alerts the Georgian National Center for Disease Control and Public Health. EIDSS detected a median of one CCHF case per year (range: 0–13 cases) from 2009 to 2013, totaling 15 cases during that time period. A case was defined as fever (temperature >100.4°F [>38°C]), one or more hemorrhagic signs (petechial or purpural rash, bleeding, or thrombocytopenia) and laboratory confirmation (i.e. a positive test for CCHF nucleic acid or anti-CCHF IgM).

From January to September 2014, the surveillance system detected 22 cases of CCHF, the highest annual case count since surveillance began in 2009 [12, 13]. Seventeen (77.3%) case-patients were residents of rural villages. EIDSS had previously only detected three cases in these same villages from 2009–2013, a CCHF prevalence of only 0.03%, based on the 2002 Georgian census reporting a population of 11,925 people in these specific villages. Most (77.3%) case-patients were able to identify a known CCHF risk factor preceding their illness, citing tick exposures and/or animal blood exposures [14]. Beyond the 22 cases detected, the extent of CCHF transmission, as well as the CCHF risk factors in the villages reporting a 2014 CCHF case, were unknown. In fact, the previously low annual CCHF case counts limited large-scale CCHF investigations in Georgia to date, rendering the overall burden of CCHF and associated risk factors in Georgia unknown. Thus, data to inform immediate public health interventions in these communities were limited. In the rural villages reporting at least one 2014 CCHF case, we launched an investigation to determine CCHF seroprevalence, identify risk factors, and document CCHF-related knowledge, attitudes, and practices (KAP). Ultimately, the goal was to use this data to direct immediate public health interventions in order to mitigate CCHF risk and transmissibility in these communities, as well as to inform future prevention and response efforts in the country of Georgia.

## Materials and Methods

**Fig 1:**
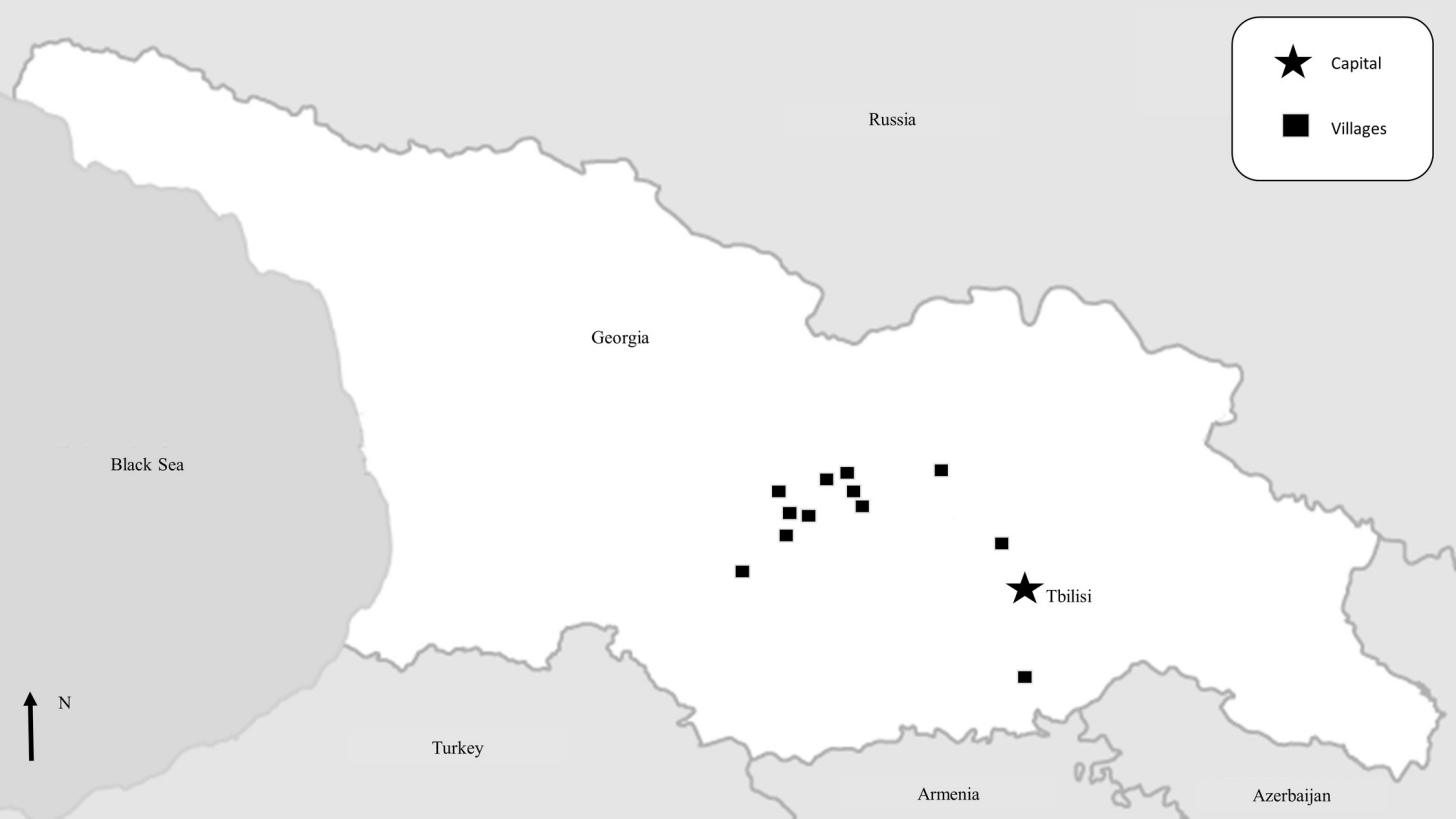
The 12 rural villages reporting at least one Crimean-Congo hemorrhagic fever (CCHF) case from January to September 2014 in Georgia. Georgia is demarcated in white with surrounding countries in gray. The capital, Tbilisi, is indicated by a star. The black squares denote the villages that had at least one CCHF case reported in the Electronic Integrated Disease Surveillance System from January to September 2014. Map adapted from MapsOpenSource.com.

We conducted a knowledge, attitudes and practices (KAP) and risk factor survey (referred to as the KAP/risk factor survey), as well as a CCHF serosurvey in the 12 affected rural villages, defined as villages with at least one CCHF case reported from January to September 2014 (Fig 1). Participants could enroll in the KAP/risk factor survey, the serosurvey, or both. The survey was administered over a one-week period in October 2014.

### Investigation Population

All available adult household members in the selected households who met the inclusion criteria were identified and enrolled. Inclusion criteria included 1) an adult (≥18 years old) member of the household who could give consent and 2) resident of the household or village for the preceding two months to ensure that any anti-CCHF IgM positivity would correspond to the selected village. Exclusion criteria included CCHF symptoms at the time of the interview, age less than 18 years, not having lived in the village or in the household for the preceding two months, and not being able to give consent.

### Sample Size

We calculated a total sample size of 905 participants or 457 households by allocating the sample size for each village proportional to the population size based on the 2002 Georgian census and on the following assumptions: 1) alpha is 0.05 (95% confidence interval), 2) CCHF seroprevalence is 2.7% ± 5%, based on data reported from endemic countries [4, 9, 10, 15–17], 3) design effect of 1, assuming minimal household clustering, 4) response rate of 90%, and 5) adult household size of at least two [18].

### Investigation Design

Using Google Earth^TM^ (Version 7.1.2.2041, Google Inc., Mountain View, CA, USA) satellite imagery of the 12 affected villages, rooftops were enumerated. Using a random number generator, we randomly selected rooftops and recorded their global positioning system (GPS) coordinates. In the field, survey teams used GPS mobile devices (2007, Garmin Ltd., Olathe, KS, USA) to locate the selected structures [19]. Each selected structure was categorized as a household, an abandoned house, a summer house (defined as a secondary house that is usually only inhabited during the summer months), or not a house (e.g. commercial property, church, barn, etc.). If the assigned rooftop was not a house, or was confirmed by neighbors to be an abandoned house or a summer house, the next proximal house to the right that met the household definition was selected.

#### KAP/risk factor survey

The KAP/risk factor survey was translated into Kartuli, the most commonly spoken language in Georgia, and then back translated into English by another translator. Any discrepancies were discussed between the two translators. Five people underwent pilot testing of the questionnaire: two had background and understanding of the daily practices in the affected villages to ensure answer choices were appropriate and culturally sensitive, while the other three’s highest education level was secondary school or less, to ensure the language of the survey could be understood at the secondary education level. Changes were made to ensure terminology was culturally appropriate, less scientific, and that the answer choices accurately reflected the daily activities in the rural villages (SI Survey). The questionnaire was also translated in Azeri for the one Azerbaijani village investigated.

The KAP/risk factor survey was administered verbally to participants based on their preferred language (Kartuli or Azeri) by interviewers fluent in the language. The survey instrument contained questions regarding demographics, CCHF risk factors, CCHF-related knowledge, attitudes, and practices, history of illness in the last four months, and symptoms of fever and hemorrhage in the last five years. At the conclusion of the interview, households received educational material regarding CCHF infection and preventive practices, as well as contact information for the local public health center.

#### Serosurvey

For participants consenting to the serosurvey, a sample of 10 ml whole blood was obtained from each willing participant for CCHF serological testing. Serologic testing was performed at the R. G. Lugar Center for Public Health Research (Tbilisi, Georgia) for recent (within the past four months) and past (within last five years) CCHF infection as demonstrated by anti-CCHF IgM and IgG, respectively [1]. Testing was performed using the commercially available CCHF virus IgM and IgG enzyme-linked immunosorbent assay (ELISA) kits (Vector-Best Company, Novosibirsk, Russia) according to manufacturer’s instructions [20]. All testing was performed in duplicate. The negative control wells’ mean optical density plus 0.2 produced the cutoff value for the assay. A positive sample was defined as an absorbance value higher than the calculated cutoff value, per manufacturer’s instructions. If any sample was found to be anti-CCHF IgM positive, reverse transcriptase-polymerase chain reaction (RT-PCR) was performed.

### Data Analysis

KAP/risk factor survey responses were de-identified and entered into a database (EpiInfo^TM^, version 7.1.1.14, CDC, Atlanta, GA, USA). Double data entry was performed by two different investigators. We randomly selected 10% of records entered in the database for review to ensure proper data entry by comparison with the original questionnaire.

Both serologic and KAP/risk factor survey data were analyzed using EpiInfo^TM^ and SAS (Version 9.3, SAS Institute Inc., Cary, NC, USA). KAP data and the overall seroprevalence calculation were weighted for non-response as well as gender by each village, using Georgia’s 2002 national census data on male to female ratios in the affected villages. Two participants had missing answers for the gender question and thus, data were imputed based on male to female ratios in other households; percentages presented reflect the weighting. The serosurvey data were analyzed unweighted.

Answers to the knowledge questions were scored as correct or incorrect, based on evidence from the literature. For risk factor analysis, high-risk activities were evaluated based on known CCHF vectors as well as previously known activities of daily living/practices in the affected villages, including: animal husbandry, herding, exposure to ticks, butchering raw meat, slaughtering, assisting in animal births, drinking unpasteurized milk, working in a healthcare setting, and involvement in agriculture [1–3, 6, 15–17, 21] (S1 Survey). Thus, a participant could be involved in a maximum of nine high-risk activities. Animal blood exposure was defined as assisting with animal births, slaughtering and/or butchering animals. Tick exposure was defined as any interaction with ticks including direct physical contact, bites, as well as exposure to ticks around the work and/or home environment.

Bivariate testing was performed using Chi-square analysis to evaluate frequencies between variables, and the Mann-Whitney U test to analyze medians. For comparison of three or more groups, the Kruskal Wallis Test was used. Significance was set at a p-value greater than 0.05.

### Ethical Review

Written informed consent was obtained from all participants. No personal identifying information was included. This investigation was reviewed in accordance with United States Centers for Disease Control and Prevention human subjects review procedures and was determined to be a non-research public health response activity.

## Results

### KAP/risk factor survey

During the one week of data collection, the investigative team visited 453 houses; occupants were not home in 26 (5.8%) houses. A total of 657 people (1.5 people per household) were approached for enrollment: 14 (2.1%) were not eligible, 17 (2.6%) refused to participate, and one (0.1%) did not complete the questionnaire. Additionally, seven questionnaires went missing in the field. Thus, we conducted a total of 618 completed interviews (1.4 participants per household).

Of the 618 participants surveyed, 215 (48.8%) were male. Median respondent age was 54.8 years (IQR 26.5, range: 18.6–101.4). Most (47.1%) participants reported an agrarian occupation. Of 617 responding, 54.6% participants’ highest completed education level was secondary school. Of 616 responding, 73.9% participants had income less than 500 Lari per month (approximately 250 US Dollars). Additionally, of 612 participants, 355 (55.8%) owned less than 2,001m^2^ of land (Table 1).

**Table 1:** Demographics of survey participants during an investigation of the 12 rural villages with reporting at least one 2014 Crimean-Congo hemorrhagic fever case from January to September 2014 in Georgia.

Characteristics	Median (IQR)		Range
Age, years (n = 613)	54.8 (26.5)		18.6–101.4
Characteristics	n	% ^†^	95% CI^ǂ^
Gender (n = 618)			
Male	215	48.8	44.6–53.1
Ethnicity (n = 613)			
Georgian	544	89.3	88.3–90.3
Azeri	55	8.7	8.1–9.4
Other	14	2.0	0.7–2.7
Occupation (n = 618)			
Agriculture	289	47.1	42.9–51.3
Housework	160	21.1	17.9–24.1
Farmer	62	11.1	8.3–13.9
Office worker	45	7.4	5.2–9.6
Herder	4	0.7	0.0–1.4
Healthcare worker	3	0.4	0.0–0.8
Veterinarian	2	0.4	0.0–0.9
Slaughterhouse worker	0	0.0	-
Butcher	0	0.0	-
Other	53	11.8	8.8–14.9
Education (n = 617)			
Elementary	36	5.6	3.7–7.4
Secondary	333	54.6	50.5–58.7
Vocational	126	20.1	16.8–23.4
Higher	117	19.1	15.8–22.3
Other	5	0.6	0.1–1.3
Monthly Income, US Dollars (n = 616)			
<50	64	10.1	7.7–12.6
50–250	453	73.9	70.3–77.5
251–500	88	14.3	11.5–17.2
>501	8	1.1	0.3–1.9
Don’t know	3	0.5	0.0–1.2
Land Ownership (n = 612)			
Rent	3	0.4	0.0–0.9
<1000m^2^	181	28.5	24.8–32.1
1000-2000m2	171	26.9	23.3–30.7
2001-3000m2	40	7.1	4.9–9.3
>3001m2	217	37.1	32.9–41.1

IQR: Interquartile range.

^†^Weighted percentage.

^ǂ^CI: Confidence interval.

Of 593 respondents, 429 (71.3%) had heard of CCHF previously. Most (81.1%) participants reported hearing about CCHF through media avenues; of these, 306 (89.1%) cited a television source. Of 429 participants responding to the knowledge questions, 348 (79.5%) were able to correctly identify at least one mode of CCHF transmission; most (74.8%) identified a tick bite. Most (80.3%) participants were able to correctly identify a CCHF risk activity, citing working with livestock as the most common (73.1%). Those with CCHF high-risk activity knowledge were more likely to be engaged in those activities than other surveyed (OR: 2.7, CI: 1.2–6.2, p = 0.0140). Additionally, most (65.7%) participants were able to correctly report at least one sign or symptom of CCHF; of those, 95.5% identified fever, 48.9% headache, and 41.2% nausea and vomiting. Of 612 responding, 184 (28.8%) knew correct protective measures against CCHF and tick exposures; of those, 102 (54.3%) employed the protective measures (Table 2).

**Table 2:** Knowledge of Crimean-Congo hemorrhagic fever (CCHF) among survey participants during an investigation of the 12 rural villages reporting at least one 2014 CCHF case from January to September 2014 in Georgia.

	All responses			Correct responses		
Knowledge	n	%*	95% CI^†^	n	%*	95% CI^†^
Transmission (n = 429)^ǂ^				348	79.5	75.4–83.5
Tick bite	330	74.8	70.4–79.2			
Crushing a tick with bare hands	120	27.8	23.4–32.2			
Infected animal blood	65	15.5	11.8–19.2			
Infected animal bodily fluids	13	3.4	1.4–5.4			
Raw meat	14	3.5	1.5–5.5			
Infected humans	4	0.9	0.0–1.8			
Unpasteurized milk	7	1.7	0.4–2.9			
Other	10	2.6	0.9–4.2			
Don’t know	74	18.5	14.6–22.3			
High-risk activities (n = 429)^ǂ^				349	80.3	76.3–84.4
Working with livestock	317	73.1	68.7–77.6			
Agriculture	203	46.3	41.4–51.2			
Slaughtering	27	7.0	4.2–9.8			
Butchering	29	7.3	4.5–10.0			
Veterinarian	4	0.9	0.0–1.9			
Healthcare	2	0.4	0.0–0.9			
Other	9	2.3	0.7–3.8			
Don’t know	78	18.9	14.9–22.9			
Signs/Symptoms (n = 429)^ǂ^				290	65.7	60.9–70.4
Fever	278	62.5	57.7–67.3			
Headache	138	32.1	27.4–36.8			
Nausea/vomiting	123	27.1	22.8–31.3			
Diarrhea	10	2.0	0.7–3.2			
Muscle Pain	33	8.6	5.6–11.6			
Joint Pain	25	5.5	3.3–7.6			
Weakness	48	9.9	7.1–12.6			
Bruising	17	4.4	2.2–6.6			
Bleeding/hemorrhage	12	2.3	1.0–3.7			
Other	44	8.8	6.2–11.5			
Don’t know	137	33.7	29.1–38.4			
Protection Measures (n = 612)				184	28.8	25.1–32.6
Yes	286	45.9	41.7–50.1			
No	82	13.1	10.4–15.9			
Don’t know	244	41.0	36.9–44.9			

*Weighted percentage.

†CI: Confidence interval.

ǂMultiple answers possible.

In terms of attitudes, 359 of 612 (57.8%) participants perceived ticks as a problem in their community. Additionally, 364/611 (58.7%) respondents stated that CCHF is a problem in their community (Table 3). Of 613 responding, 56.8% were concerned about contracting CCHF but were equally likely to be engaged in CCHF high-risk activities compared with the 19.3% who were not concerned (p = 0.5302).

**Table 3:** Attitudes regarding Crimean-Congo hemorrhagic fever (CCHF) and ticks among survey participants during an investigation of the 12 rural villages reporting at least one 2014 CCHF case from January to September 2014 in Georgia.

Attitude	n	%*	95% CI^†^
People frequently get tick bites in the community (n = 614)			
Yes	199	31.7	28.1–35.3
No	165	26.9	23.2–30.5
Don't know	250	41.4	37.4–45.4
Ticks are a problem in the community (n = 612)			
Yes	359	57.8	53.9–61.7
No	87	15.8	12.7–18.9
Don't know	166	26.4	22.9–29.8
CCHF is a problem in the community (n = 611)			
Yes	364	58.7	54.8–62.6
No	57	9.8	7.3–12.3
Don't know	190	31.5	27.8–35.2
CCHF is something I am worried about (n = 613)			
Yes	355	56.8	52.8–60.8
No	114	19.3	16.1–22.6
Don't know	144	23.9	20.4–27.4

*Weighted percentage.

†CI: Confidence interval.

Of 618 participants responding, 565 (91.5%) reported involvement in at least one known CCHF high-risk activity (median: 2.6 activities, IQR 0.1, range: 0–7); there was no difference among gender (p = 0.5299). Of 605 respondents, 477 (79.3%) reported involvement in agriculture. Additionally, 434 (69.9%) respondents reported participation in animal husbandry; of those, 339 (78.6%) owned cattle. Of 389 (63.3%) participants who reported tick exposures, 286 (46.7%) handled ticks bare-handed; 65 (29.7%) of 216 answering knew the associated risk. Of 605 respondents, 355 (57.9%) reported animal blood exposures; 32 (12.7%) of 281 answering knew the associated risk. Of 565 responding, 65 (13.7%) were involved in animal births; of those, 36 (55.6%) did not use personal protective equipment (Table 4).

**Table 4:** Crimean-Congo hemorrhagic fever (CCHF) high-risk activities among survey participants during an investigation of the 12 rural villages reporting at least one 2014 CCHF case from January to September 2014 in Georgia.

High-Risk Activities	n	%*	95% CI^†^
Agriculture (n = 605)	477	79.3	75.9–82.8
Animal husbandry (n = 618)	434	69.9	66.3–73.6
Tick exposure (n = 618)	389	63.3	59.5–67.1
Butchering raw meat (n = 603)	334	53.4	49.3–57.5
Herding (n = 585)	102	20.3	16.7–23.8
Animal births (n = 565)	65	13.7	10.5–16.9
Slaughtering (n = 603)	42	7.9	5.5–10.2
Drinking unpasteurized milk (n = 602)	36	5.9	4.0–7.7
Healthcare settings (n = 604)	2	0.3	0.0–0.7

*Weighted percentage.

†CI: Confidence interval.

In terms of CCHF preventive practices, 339/599 (57.3%) respondents denied employing personal protective measures against CCHF and tick exposures. Of the 202 who knew and employed personal protective measures, the most common method (84.5%) was using personal protective equipment, defined as wearing long, covered clothing and gloves. Of 404 responding, 370 (92.1%) employed a protective measure to prevent ticks for their animals; 83.4% used insecticides. For tick removal, of the 324 participants who removed ticks from their animals, 111 (35.0%) removed the ticks using bare hands. Of the 234 respondents that reported having ticks on their body, 193 (81.1%) also removed the ticks with bare hands (Table 5).

**Table 5:** Tick prevention and removal practices among survey participants during an investigation of the 12 rural villages reporting at least one 2014 Crimean-Congo hemorrhagic fever case from January to September 2014 in Georgia.

Human-tick interaction	n	%*	95% CI^†^
Method to prevent (n = 599)^ǂ^			
Nothing	339	57.3	53.2–61.3
Personal protective equipment^§^	172	28.0	24.3–31.7
Pesticides	25	4.9	2.9–6.9
Repellent	10	1.7	0.6–2.8
Avoid woody/rural areas	8	1.5	0.4–2.6
Other	15	2.5	1.4–4.1
Don't know	58	9.6	7.2–11.9
Method to Remove Ticks (n = 234)			
Bare hands	193	81.8	76.4–87.3
Use an object	21	8.6	4.7–12.4
Gloves	5	2.3	0.2–4.4
Hospital	2	0.7	0.0–1.6
Other	13	6.7	2.9–10.4
Animal-tick interaction	n	%*	95% CI^†^
Method to prevent (n = 404)^ǂ^			
Insecticides	338	83.4	75.5–87.2
Nothing	34	7.9	5.3–10.6
Injectable	20	5.6	3.1–7.9
Other	25	6.4	3.8–8.9
Method to remove ticks (n = 324)			
Pour insecticides	184	56.5	50.8–62.2
Bare hands	111	35.0	29.5–40.5
Object	20	5.8	3.2–8.5
Veterinarian	3	1.0	0.0–1.9
Other	6	1.7	0.3–3.1

*Weighted percentage.

†CI: Confidence interval.

ǂMultiple answers possible.

§Includes wearing long, covered clothing and gloves.

### Serosurvey

Of 643 people approached and eligible, 444 (69.1%) consented to the CCHF serosurvey. Of the 110 participants that provided a reason for refusal, 63 (57.3%) stated they were “too scared” of the needle. Additionally, five participants were unable to provide a sample due to poor venous access. Thus, 439 samples underwent laboratory analysis (0.9 samples per household). Of these, one participant consented for blood and not the survey, one survey was missing in the field, and two blood specimens were mislabeled. As such, survey data were available for 435 participants for analysis.

In total, 12 (2.8%) samples were anti-CCHF IgG positive, indicating past infection within four months to five years prior, and one (0.2%) was anti-CCHF IgM positive, indicating recent infection (1,2). On further examination, the anti-CCHF IgM positive participant was asymptomatic, as well as RT-PCR and anti-CCHF IgG negative. The participant remained asymptomatic and had similar laboratory results on repeat testing four months after initial testing, indicating a false-positive. Thus, only the 12 anti-CCHF IgG positive cases underwent analysis. When controlled for gender and non-response, the seroprevalence was weighted to 3.0% (CI: 1.2–4.8).

**Fig 2:**
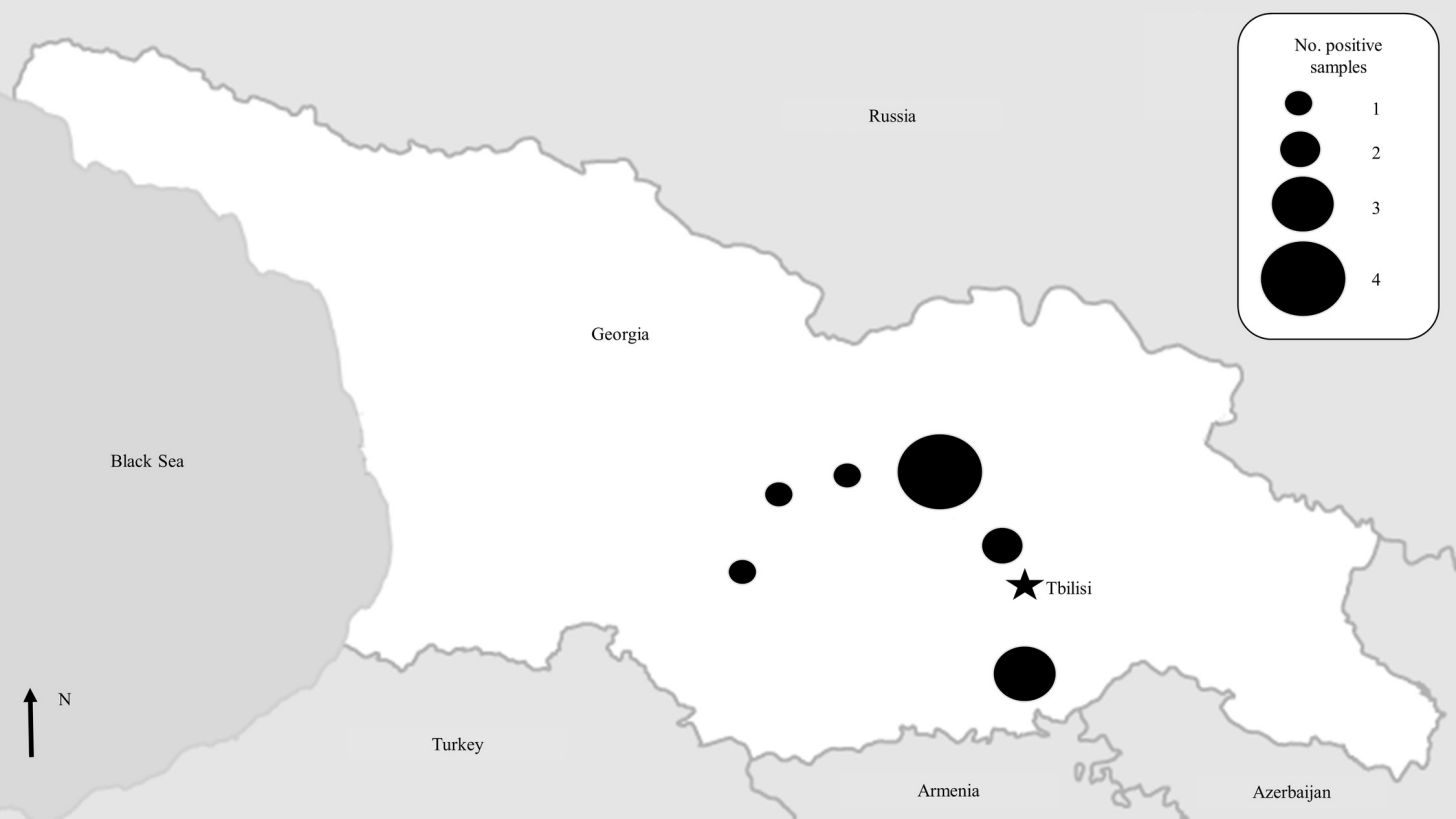
Geographic distribution of Crimean-Congo hemorrhagic fever (CCHF) seropositive participants among survey participants during an investigation of the 12 rural villages reporting at least one 2014 CCHF case from January to September 2014 in Georgia. Georgia is demarcated in white with surrounding countries in gray. The capital, Tbilisi, is indicated by a star. Circle size indicates the number of seropositive subjects in that area; the bigger the circle, the higher the number of seropositive subjects. Map adapted from MapsOpenSource.com.

The seropositive subjects were located in six (50.0%) of the 12 villages tested (Fig 2). Seven (58.3%) seropositive participants were male. Median seropositive subjects’ age was 71.2 years (IQR: 18.3, range: 49.8–84.9). Most (66.6%) reported an agrarian occupation (Table 6).

**Table 6:** Crimean-Congo hemorrhagic fever (CCHF) seropositive subjects’ demographics among survey participants during an investigation of the 12 rural villages reporting at least one 2014 CCHF case from January to September 2014 in Georgia (N = 12).

Characteristics	Median (IQR)		Range
Age, years	71.2 (18.3)		49.8–84.9
Characteristics	n	%	95% CI^†^
Gender			
Male	7	58.3	27.7–84.8
Ethnicity			
Georgian	9	75.0	42.8–94.5
Azeri	2	16.7	2.1–48.4
Other	1	8.3	0.2–38.5
Occupation			
Gardening	6	50.0	21.1–78.9
Housework	2	16.7	2.1–48.4
Farmer	1	8.3	0.2–38.5
Herder	1	8.3	0.2–38.5
Office	0	0.0	-
Slaughterhouse Worker	0	0.0	-
Butcher	0	0.0	-
Healthcare Worker	0	0.0	-
Veterinarian	0	0.0	-
Other	2	16.7	2.1–48.4
Education			
Elementary	4	33.3	9.9–65.1
Secondary	4	33.3	9.9–65.1
Vocational	3	25.0	5.5–57.2
Higher	1	8.3	0.2–38.5
Other	0	0.0	-
Monthly Income, US Dollars			
<50	1	8.3	0.2–38.5
51–250	10	83.3	51.6–97.9
251–500	1	8.3	0.2–38.5
>501	0	0.0	-
Land Ownership			
Rent	0	0.0	-
<1000m^2^	0	0.0	-
1000-2000m2	5	41.7	15.2–72.3
2001-3000m2	1	8.3	0.2–38.5
>3001m2	6	50.0	21.1–78.9

IQR: Interquartile range.

†CI: Confidence interval.

Seropositive subject’s most common CCHF high-risk activity was tick exposure (66.7%); there was no significant difference compared to seronegative subjects’ tick exposure (OR: 1.0, CI: 0.3–3.4, p = 0.7696). Other CCHF high-risk activities included agriculture (58.3%), animal husbandry (58.3%), and herding (33.3%). Additionally, there was no difference in the median of high-risk activities among the seropositive subjects (median = 3.0 activities, IQR: 4, range: 0–6) compared to the seronegative subjects (median = 3.0 activities, IQR: 2, range: 0–7, p = 0.8257).

In the four months preceding the survey, four (33.3%) seropositive subjects reported fever and muscle pain; of those, one also reported concomitant jaundice and one reported concomitant bruising. None of the seropositive subjects reported symptoms during the prior four months to five years’ timeframe.

## Discussion

In response to the high CCHF case count detected in 2014, this investigation identified and characterized the CCHF risk factors, gaps in CCHF-related knowledge and practices, as well as the prevalence of the disease in the communities reporting 2014 CCHF cases, to inform immediate public health interventions. Overall, participants in all villages were found to be at high-risk for CCHF transmission, as most were engaged in known CCHF high-risk activities. More specifically, ticks were found to be a common high-risk exposure in these villages through activities associated with agriculture and animal husbandry.

Most participants heard of CCHF before the investigation, which translated into knowledge regarding CCHF transmission, CCHF high-risk activities, and CCHF-related signs and symptoms, but gaps in knowledge were still evident. Although most were aware that a tick bite was a means of CCHF transmission, very few knew that crushing a tick with bare hands was also a risk. Even those participants who engaged in high-risk activities, specifically those who handled ticks bare handed and had animal blood exposures, were unaware of the associated CCHF risk. Thus, it is important that educational campaigns are employed in these villages, targeting the specific sub-groups that are engaged in theses high-risk activities, including herders, agricultural workers, slaughterhouse workers, and veterinarians.

In addition to lack of knowledge regarding CCHF high-risk activities, the data revealed a major gap in the knowledge of and employment of personal protective measures against CCHF. Only a few participants correctly knew CCHF protective measures and of those, only half actually employed the measures. This indicates that knowledge did not translate into risk avoidance in these communities; although likely multifactorial in nature, this may be due to a reliance on these high-risk activities for livelihoods. As personal protection knowledge is lacking in these communities, as well as the employment of these measures, this topic should be highlighted in educational campaigns targeting these communities. Additionally, although risk factor avoidance should be discussed as a possible intervention, educational campaigns should highlight the employment of protection methods, as avoidance may not be possible in these communities.

The serosurvey revealed a 3.0% weighted seroprevalence of past CCHF exposure in these villages, which is consistent with reports in neighboring countries [4, 9, 10, 15–17]. Additionally, the data revealed possible subclinical cases of CCHF, as most did not report any symptoms consistent with CCHF in the previous five years, corresponding to the time when anti-CCHF IgG remains detectable [1,2]. This is consistent with findings from a previous study documenting that only about one of five people infected with CCHF develop symptoms [3].

Although the surveillance system, EIDSS, uses anti-CCHF IgM to confirm acute infection and this investigation found positive anti-CCHF IgG subjects, demonstrating infection sometime within the last five years, comparisons can still be made; EIDSS had been operating for five years prior to this investigation, within the limit of the anti-CCHF IgG seropositivity; thus, the surveillance system had the opportunity to capture these cases. Additionally, both EIDSS and this investigation used the same ELISA commercial kits ensuring consistency of the specificity and sensitivity of the testing assay used.

Overall, the seroprevalence in these villages is higher than what had been previously detected, 0.03%, by the surveillance system. Two factors likely underlie this discrepancy: one is the existence of subclinical cases, as most seropositive subjects did not report any symptoms associated with CCHF, and thus, would not be captured in EIDSS. Second, there may be issues with physicians’ recognition of CCHF signs/symptoms, as the four seropositive participants who had symptoms consistent with CCHF were not captured in EIDSS. These findings highlight the importance of continuing to improve physicians’ awareness of milder presentations of CCHF in these communities. Additionally, this underreporting will need to be considered when reporting CCHF in Georgia in the future.

There were a number of limitations to this investigation. First, summer homes were not included in this evaluation and thus, data cannot be extrapolated to this population. Nevertheless, this population may also have similar risk factors as they likely populate the homes during months with known increased tick activity, which should be considered when timing future public health interventions [22]. Second, data from this investigation can only be extrapolated to the population in the villages surveyed, not the country of Georgia. As this was an emergency investigation of the villages reporting CCHF cases in 2014 to dictate immediate public health interventions in these specific villages, other villages were not surveyed as controls. Thus, although these results cannot be generalized to other villages in Georgia, it raises the question whether this high prevalence of CCHF high-risk activities and seropositivity is unique to these investigated villages or whether these findings exist beyond the village borders. As these data trends may occur in other villages with similar characteristics and demographic profiles (located along herding routes and/or where agrarian activities are the mainstay of livelihood), it is important to consider expansion of previous educational campaigns to other similar villages. Third, females were overrepresented in the survey population. It is possible that this is a result of conducting the survey during daylight hours when men were more likely to be engaged in agricultural activities outside of the home. In an effort to address this limitation, we weighted our data analysis by gender. Fourth, the evaluation of clinical symptoms of the seropositive participants may be limited due to recall bias. Therefore, there may have been symptoms at the time of a respondent’s infection with the CCHF virus that were not remembered during survey administration. And finally, analysis of the serosurvey data was limited as the sample size goal was not obtained. Thus, there may be significant trends, including differences in exposures to high-risk activities between the seropositive subjects and seronegative subjects that are not revealed in this investigation due to insufficient power.

Even in light of these limitations, this investigation identified large gaps in CCHF-related knowledge and the use of preventive practices in the setting of a high prevalence of CCHF high-risk activities and seropositivity in these villages; these findings are critical to informing public health interventions in these communities, specifically the importance of employing educational campaigns tailored to the gaps highlighted in this investigation, targeting both community members as well as physicians. Additionally, the identification of a higher CCHF seroprevalence in these villages than previously detected highlights the importance of supporting ongoing CCHF investigations in Georgia, as well as continuing efforts to improve the surveillance system’s sensitivity. Finally, although the investigation only took place in specific villages in the country of Georgia, this is the first large scale CCHF investigation in the country. Thus, this investigation not only contributes to the previously limited knowledge regarding CCHF in the country of Georgia, but also contributes to the larger understanding of the overall regional prevalence of the disease.

### Disclaimer

The opinions expressed by authors contributing to this journal do not necessarily reflect the official position of the Centers for Disease Control and Prevention or the institutions with which the authors are affiliated.

## Supporting Information

S1 Survey Crimean-Congo Hemorrhagic Fever Knowledge, Attitudes, and Practices Survey. (PDF)

## References

[1] Ergonul O. Crimean-Congo haemorrhagic fever. Lancet Infect Dis. 2006 4;6(4):203–14. 16554245 10.1016/S1473-3099(06)70435-2 PMC7185836

[2] Appannanavar SB, Mishra B. An update on crimean congo hemorrhagic fever. J Glob Infect Dis. 2011 7;3(3):285–92. 10.4103/0974-777X.83537 21887063 PMC3162818

[3] Goldfarb LG, Chumakov MP, Myskin AA, Kondratenko VF, Reznikova OY. An epidemiological model of Crimean hemorrhagic fever. Am J Trop Med Hyg. 1980 3;29(2):260–4. 7369445 10.4269/ajtmh.1980.29.260

[4] Yilmaz GR, Buzgan T, Irmak H, Safran A, Uzun R, Cevik MA, et al The epidemiology of Crimean-Congo hemorrhagic fever in Turkey, 2002–2007. International Journal of Infectious Diseases. 2009 5;13(3):380–6. 10.1016/j.ijid.2008.07.021 18986819

[5] Knust B, Medetov ZB, Kyraubayev KB, Bumburidi Y, Erickson BR, MacNeil A, et al Crimean-Congo hemorrhagic fever, Kazakhstan, 2009–2010. Emerg Infect Dis. 2012 4;18(4):643–5. 10.3201/eid1804.111503 22469505 PMC3309686

[6] Centers for Disease Control and Prevention. Crimean-Congo hemorrhagic fever. 2014 May 9 [cited 2014 September 5]. Available: http://www.cdc.gov/vhf/crimean-congo/.

[7] World Health Organization. Crimean-Congo haemorrhagic fever: WHO; 2013 Jan [cited 2014 September 5]. Available: http://www.who.int/mediacentre/factsheets/fs208/en/.

[8] National Institute of Allergy and Infecitous Diseases [NIAID]. NIAID Biodefense Research. 2014 Aug 8 [cited 2014 September 9]. Available: http://www.niaid.nih.gov/topics/biodefenserelated/biodefense/pages/cata.aspx.

[9] Gergova I, Kamarinchev B. Seroprevalence of Crimean-Congo hemorrhagic fever in southeastern Bulgaria. Jpn J Infect Dis. 2014;67(5):397–8. 25241694 10.7883/yoken.67.397

[10] Gergova I, Kamarinchev B. Comparison of the prevalence of Crimean-Congo hemorrhagic fever virus in endemic and non-endemic Bulgarian locations. J Vector Borne Dis. 2013 12;50(4):265–70. 24499848

[11] Central Intelligence Agency. The World Factbook: Georgia. 2014 [cited 2014 September 5]. Available: https://www.cia.gov/library/publications/the-world-factbook/geos/gg.html

[12] Kuchuloria T, Imnadze P, Chokheli M, Tsertsvadze T, Endeladze M, Mshvidobadze K, et al Viral hemorrhagic fever cases in the country of Georgia: Acute Febrile Illness Surveillance Study results. Am J Trop Med Hyg. 2014;91(2):246–8. 10.4269/ajtmh.13-0460 24891463 PMC4125244

[13] Zakhashvili K, Tsertsvadze N, Chikviladze T, Jghenti E, Bekaia M, Kuchuloria T, et al Crimean-Congo hemorrhagic fever in man, Republic of Georgia, 2009. Emerg Infect Dis. 2010;16(8):1326–8. 10.3201/eid1608.100097 20678341 PMC3298293

[14] Greiner AL, Mamuchishvili N, Salyer SJ, Stauffer K, Geleishvili M, Zakhashvili K, et al Notes from the Field: Increase in Reported Crimean-Congo Hemorrhagic Fever Cases—Country of Georgia, 2014. Morbidity and Mortality Weekly Report. 2015;64(8):228–9. 25742385 PMC4584721

[15] Sharifi-Mood B, Metanat M, Alavi-Naini R. Prevalence of crimean-congo hemorrhagic Fever among high risk human groups. Int J High Risk Behav Addict. 2014;3(1):e11520 10.5812/ijhrba.11520 24971294 PMC4070186

[16] Andriamandimby SF, Marianneau P, Rafisandratantsoa JT, Rollin PE, Heraud JM, Tordo N, et al Crimean-Congo hemorrhagic fever serosurvey in at-risk professionals, Madagascar, 2008 and 2009. J Clin Virol. 2011;52(4):370–2. 10.1016/j.jcv.2011.08.008 21889395

[17] Sargianou M, Panos G, Tsatsaris A, Gogos C, Papa A. Crimean-Congo hemorrhagic fever: seroprevalence and risk factors among humans in Achaia, western Greece. Int J Infect Dis. 2013;17(12):e1160–5. 10.1016/j.ijid.2013.07.015 24084247

[18] Kish L. (1965). Survey Sampling, New York: John Wiley & Sons, Inc.

[19] Taylor TH, Jr, Zhang X, Mansour A, Deutscher M, Seitz A, Dueger E, et al. ArcGIS and Google Earth High-resolution Imagery for a Health Utilization Survey in Damanhur, Egypt [abstract]. International Conference on Emerging Infectious Diseases 2010: slide sessions and poster abstracts. Emerg Infect Dis. 2010 Jul [cited 2015 December 23]. Available: http://wwwnc.cdc.gov/eid/pages/2010-international-conference-on-emerging-infectious-diseases-iceid.htm.

[20] Vanhomwegen J, Alves MJ, Zupanc TA, Bino S, Chinikar S, Karlberg H, et al Diagnostic assays for Crimean-Congo hemorrhagic fever. Emerg Infect Dis. 2012;18(12):1958–65. 10.3201/eid1812.120710 23171700 PMC3557897

[21] Ergonul O, Celikbas A, Baykam N, Eren S, Dokuzoguz B. Analysis of risk-factors among patients with Crimean-Congo haemorrhagic fever virus infection: severity criteria revisited. Clin Microbiol Infect. 2006;12(6):551–4. 16700704 10.1111/j.1469-0691.2006.01445.x

[22] European Centre for Disease Prevention and Control.Hyalomma marginatum: Epidemiology and transmission of pathogens. 2014 [cited 2016 May 25]. Available: http://ecdc.europa.eu/en/healthtopics/vectors/ticks/Pages/hyalomma-marginatum-.aspx.

