# Supplementary material for: Crimean-Congo Hemorrhagic Fever Knowledge, Attitudes, Practices, Risk Factors, and Seroprevalence in Rural Georgian Villages with Known Transmission in 2014
Source: PLoS One. 2016 Jun 23;11(6):e0158049. doi: 10.1371/journal.pone.0158049 (PMC4918973; doi:10.1371/journal.pone.0158049)
Supplement: S1 Survey — (PDF) [file pone.0158049.s001.pdf]

Form Approved
OMB No. 0920-1011
Exp. Date 3/31/2017

Crimean-Congo Hemorrhagic Fever

Knowledge, Attitudes, and Practices Survey

October 2014
Tbilisi, Georgia

LABEL

Public reporting burden of this collection of information is estimated to average 30 minutes per response, including the time for reviewing instructions, searching existing data sources, gathering and maintaining the data needed, and completing and reviewing the collection of information. An agency may not conduct or sponsor, and a person is not required to respond to a collection of information unless it displays a currently valid OMB control number. Send comments regarding this burden estimate or any other aspect of this collection of information including suggestions for reducing this burden to CDC/ATSDR Reports Clearance Officer; 1600 Clifton Road NE, MS D-74 Atlanta, Georgia 30333; ATTN: PRA (0920-1011)

OMB No. 0920-1011
Exp. Date 3/31/2017

SURVEY

LABEL

Introduction

Note: When administering the following survey, do not prompt any of the multiple choice answers; please have the participant state their own answers.

To the interviewee: "Thank you for being willing to participate in this survey. I am going to start by asking you basic questions about yourself to get to know you better. Please note that your name and any other identifying information will not be collected during this survey. If you want to have the survey stopped at any time or for any reason, please tell us immediately."

Demographics

1. Date of birth (DD/MM/YYYY): _______________
2. Sex:
    a. Male
    b. Female
3. Nationality:
    a. Georgian
    b. Azery
    c. Armenian
    d. Other: ____________
4. Residence:
    a. Rural
    b. Urban
5. Household Size (including the participant): ____________
6. Are you registered to vote?
    a. Yes
    b. No
7. Highest education level: (one answer only)
    a. Elementary
    b. Secondary
    c. Vocational
    d. Higher
    e. None

f. Other: ______________

8. What daily activity do you perform for greater than 6 hours a day? (circle one answer only)
    a. Farmer
    b. Herder
    c. Gardening/Work in the Field
    d. Slaughterhouse worker
    e. Butcher
    f. Healthcare worker
    g. Veterinarian
    h. Work in an office
    i. Other____________________
9. What is your monthly household income?
    a. <100 Lari
    b. 100-500 Lari
    c. 501-1000 Lari
    d. 1001-1500 Lari
    e. 1501-2000 Lari
    f. 2001-3000 Lari
    g. >3001 Lari
    h. I don't know
10. Do you receive social security?
    a. Yes
    b. No
11. How much land do you own?
    a. I rent the land
    b. $<1000m^2$
    c. $1000-2000m^2$
    d. $2001-3000m^2$
    e. $3001-4000m^2$
    f. $4001-8000m^2$
    g. $>8000m^2$

Risk Factors

12. Do you own or take care of animals? (circle all the apply)
    a. No
    b. If yes, what type?
        i. Sheep
        ii. Goats
        iii. Cattle
        iv. Buffalo
        v. Chickens
        vi. Horses
        vii. Donkeys

viii. Other ________________

13. In the last four months, have you performed the following activities (circle all the apply):
    a. Herding
        i. No
        ii. Sheep
        iii. Goats
        iv. Cattle
        v. Buffalo
        vi. Other________________________
    b. Have you assisted an animal birth?
        i. Have assisted in animal birthing but have used PPE (gloves, gowns, boots)
        ii. Have assisted in animal birthing but have **not** used PPE
        iii. Have not assisted in animal birthing
    c. Slaughtering
        i. No
        ii. Sheep
            1. Slaughter sheep using PPE (gloves, gowns, boots)
            2. Slaughter sheep without PPE (gloves, gowns, boots)
        iii. Goats
            1. Slaughter goats using PPE (gloves, gowns, boots)
            2. Slaughter goats without PPE (gloves, gowns, boots)
        iv. Cattle
            1. Slaughter cattle using PPE (gloves, gowns, boots)
            2. Slaughter cattle without PPE (gloves, gowns, boots)
        v. Buffalo
            1. Slaughter buffalo using PPE (gloves, gowns, boots)
            2. Slaughter buffalo without PPE (gloves, gowns, boots)
        vi. Other________________________
            1. Slaughter animals using PPE (gloves, gowns, boots)
            2. Slaughter animals without PPE (gloves, gowns, boots)
    d. Butchering/handling raw meat
        i. No
        ii. Sheep
        iii. Goats
        iv. Cattle
        v. Buffalo
        vi. Other________________________
    e. Handled ticks with bare hands
        i. No
        ii. Removed ticks from animal and threw is out
        iii. Removed ticks from animals and killed with bare hands
        iv. Removed ticks from yourself and threw it out

v. Removed ticks from yourself and killed with bare hands
vi. Other____

f. Worked in a health care setting
   i. No
   ii. Primary healthcare
   iii. Clinic
   iv. Hospital
   v. Other______________________
g. Drank unpasteurized milk
   i. Yes
   ii. No
h. Gardening
   i. Yes
   ii. No
i. Any other outdoor activity not previously asked:
   i. None
   ii. Hiking
   iii. Camping
   iv. Hunting
   v. Fishing
   vi. Picnicking outside
   vii. Other________________

14. In the last four months, have you had a tick bite?
   a. No
   b. Yes, describe each situation:

Date of Tick Bite (MM/YYYY)	Where? (village/rayon/region)	Where? (body location)	How much time did it take to get it removed after it was found?

15. Any travel or migration outside your rayon in the last four months?
   a. No
   b. Yes, describe:

Location (village/rayon/region)	Reason	Dates

16. Were you visited by the household educational campaign last few months?
   a. Yes

b. No
c. I don’t remember

KAP Information

Reminder: When administering the survey, do not prompt any of the multiple choice answers; please have the participant state their own answers.

To the interviewee: “Now I am going to ask you questions regarding what you know about Crimean-Congo Hemorrhagic Fever and what you do to protect yourself and your animals.”

Knowledge

17. Have you ever heard about Crimean-Congo Hemorrhagic Fever, also known as CCHF?
    a. Yes (proceed to question 2)
    b. No (proceed to **Attitudes section**)
    c. I don’t know
18. Where have you learned/heard about CCHF? (circle all that apply)
    a. School
    b. Media
        i. TV
        ii. Radio
        iii. Newspaper/Magazines
        iv. Pamphlets
            1. Where did you receive it? ___________________
        v. Posters
            1. Where did you see it? ___________________
    c. Educational campaign last few months (July-October)
    d. Training courses
    e. Health care worker
    f. Know someone who had CCHF
        a. Who? _____________
    g. I don’t know
    h. Other______________________
19. What are ways in which a human can become infected? (circle all that apply)
    a. Bite from a tick
    b. Crushing a tick with bare hands
    c. Contact with blood from infected animals
    d. Contact with birthing tissues/fluids from infected animals
    e. Eating raw, infected meat
    f. Contact with blood from people sick from CCHF
    g. Drinking unpasteurized milk
    h. I don’t know

   i. Other ______________________

20. What activities can put you at risk of getting the disease? (circle all that apply)
   a. Working with livestock
   b. Working in produce/vegetable/grain fields
   c. Working in the garden
   d. Working in a rural, woody area
   e. Slaughtering animals
   f. Butchering meat
   g. Working in a hospital
   h. Being a abattoir/slaughterhouse worker
   i. Working as a veterinarian
   j. Working as a health care worker
   k. I don't know
   l. Other_______________________
21. What are the signs and symptoms of CCHF? (circle all that apply)
   a. Fever
   b. Headache
   c. Nausea/Vomiting
   d. Diarrhea
   e. Muscle pain
   f. Joint pain
   g. Weakness
   h. Cough
   i. Blood in the urine
   j. Blood in the stool (black or bright red)
   k. Coughing blood
   l. Red eyes
   m. I don't know
   n. Other__________________

Attitudes

22. Do people frequently get bitten by ticks in your community?
   a. Yes
   b. No
   c. I don't know
23. Do you think ticks are a problem in your community?
   a. Yes
   b. No
   c. I don't know
24. Do you think there are more ticks this year than previously?
   a. Yes
   b. No

   c. I don’t know
25. Do you think CCHF is a problem in your community?
   a. Yes
   b. No
   c. I don’t know
26. Do you think CCHF is something you should be worried about?
   a. Yes
   b. No
   c. I don’t know
27. Do you think you can protect yourself from CCHF?
   a. Yes
      i. How? _________________
   b. No
   c. I don’t know

Practices

28. Do you have any interaction with ticks during your **job**?
   a. Yes
      i. Please describe_________________
   b. No
29. Do you have any interaction with ticks at **home**?
   a. Yes
      i. Please describe__________________
   b. No
30. If you interact with ticks, what method do you use to remove ticks off **yourself**? (circle only one answer)
   a. Remove by hand
   b. Remove with tweezers
   c. Go to a hospital/health care center
   d. I don’t interact with ticks
   e. I don’t remove ticks
   f. Other_________________
31. What do you do to protect **yourself** from ticks/CCHF? (circle all that apply)
   a. Protective clothing (i.e. long pants, socks, etc.)
      i. How often? Always Sometimes Never
   b. Treat your clothing with repellent
      i. How often? Always Sometimes Never
   c. Insect repellent on yourself
      i. How often? Always Sometimes Never
   d. Use pesticides in the environment
      i. How often? Always Sometimes Never
   e. Avoid woody/rural areas

i. How often? Always Sometimes Never

f. Nothing
g. I don't know
h. Other_________________
    i. How often? Always Sometimes Never

32. What care would you seek, if any, if you experienced symptoms of CCHF (fever, muscle aches, nausea/vomiting, bloody stools or urine, etc.)? (circle one answer only)
    a. Go to a hospital/healthcare facility
        i. Primary healthcare
        ii. District
        iii. Regional
        iv. Tbilisi ID hospital (IPC)
        v. Any other clinic in Tbilisi:______________
        vi. Other:_________________
    b. Stay at home
    c. Try local pharmacy
    d. Go to a local healer
    e. Nothing
    f. Other_________________

The following questions refer to livestock; if the participant said NO to **Question 12**, skip to the question below and proceed to the **Educational Campaign** section.

33. How do you prevent ticks for your animals? (circle all that apply)
    a. Use insecticides/acaricide
        i. Spray
        ii. Pour on
        iii. Other_______________
    b. Injectable medication
    c. Nothing
    d. Other________________
34. What method do you use to remove ticks off your livestock? (circle one answer only)
    a. Remove by hand
    b. Remove with tweezers
    c. Go to a veterinarian
    d. Pour liquid/mixture onto the tick/animal
        What kind?
        i. Oil
        ii. Alcohol
        iii. Insecticide
        iv. Other______________
    e. There's never been a tick on my animal(s)

f. Nothing
g. Other________________

Educational Campaign

Note: If the participant answered no to **Question 16** and/or is not from the following regions, skip this section and proceed to the **Past Illness** section.

Please check which one applies:

- ☐ Samtskhe-Javakheti Region
  - Borjomi
- ☐ Shida Kartli Region
  - Khashrui
- ☐ Shida Kartli Region
  - Kreli, Gori, Kaspi

To the interviewee: "Now I am going to ask you questions about the educational campaign that was performed recently regarding Crimean-Congo Hemorrhagic Fever."

35. Has your understanding of CCHF changed since the educational campaign? (circle all the apply)
    a. Yes
        i. I understand how CCHF is transmitted
        ii. I understand the signs and symptoms
        iii. I know ways to protect myself/others
        iv. Other____________________
    b. No
        i. The information was not useful
        ii. I didn't understand the information
        iii. I already knew all about CCHF
        iv. Other___________________
    c. I don't know
36. Has your perception of CCHF changed since the educational campaign? (circle all that apply)
    a. Yes
        i. I am more aware of CCHF
        ii. I am aware this is a problem in the community
        iii. I am aware this is a problem in Georgia
        iv. I believe protective equipment/procedures are important
        v. I am aware that CCHF can be dangerous
        vi. I am concerned about my safety
        vii. I am concerned about my family/community's safety
        viii. I am concerned about my job

      ix. Other____________________
   b. No
      i. The information was not useful
      ii. I didn't understand the information
      iii. I already knew all about CCHF
      iv. Other__________________
   c. I don't know
37. Has the way you protect yourself changed since the educational campaign? (circle all that apply)
   a. Yes
      i. I wear long shirts/long pants
      ii. I use repellent
      iii. I use insecticides
      iv. I avoid outdoor/woody areas
      v. Other__________________
   b. No
      i. The information was not useful
      ii. I didn't understand the information
      iii. I already knew how to protect myself
      iv. I don't like wearing protective clothing
      v. I don't like using repellent
      vi. I don't like using insecticides
      vii. Other__________________
   c. I don't know
38. Has the way you interact with ticks for both yourself and livestock changed since the educational campaign? (circle all that apply)
   a. Yes
      i. I don't handle ticks with my bare skin
      ii. I remove ticks immediately
      iii. I use repellent
      iv. I use insecticides
      v. I use injections
      vi. I consult a healthcare worker
      vii. I consult the veterinarian
      viii. Other_______________
   b. No
      i. The information was not useful
      ii. I didn't understand the information
      iii. I already knew how to handle ticks properly
      iv. Other_________________
   c. I don't know

Past Illness

39. Have you ever been diagnosed with CCHF?
    a. No
    b. If yes, describe:
        i. Date:
        ii. Where were you diagnosed:
        iii. What symptoms did you have (choose all answers that apply)?
            a. Fever
            b. Headache
            c. Nausea/Vomiting
            d. Diarrhea
            e. Muscle pain
            f. Weakness
            g. Cough
            h. Blood in the urine
            i. Bloody or black stools
            j. Coughing blood
            k. Bleeding from the gums
            l. Other_________________

To the interviewee: "Now I am going to ask about any illnesses you might have had in the last five years"

40. Have you ever had both fever and hemorrhaging at the same time in the last 5 years?
    a. No (Skip question 41, and go to question 42)
    b. Yes
        iv. What Date ______________________
        v. What Symptoms (choose all answers that apply):
            a. Fever
            b. Headache
            c. Nausea/Vomiting
            d. Diarrhea
            e. Muscle pain
            f. Weakness
            g. Cough
            h. Blood in the urine
            i. Bloody or black stools
            j. Coughing blood
            k. Bleeding from the gums
            l. Other_________________
41. Did you seek any care for your symptoms?

a. Yes
   i. Where?_______________
   ii. When?________________
b. No
   i. Why not? ______________________

Recent Illness

To the interviewee: "Now I am going to ask about any illnesses you might have had during the past four months"

42. Have you had any illness in the last four months?
    a. Yes
    b. No (End questionnaire)

43. What are dates for each illness you had in the last four months? (show calendar)

Date Started (DD/MM/YYYY)	Date Ended (DD/MM/YYYY)
1.	
2.	
3.	

44. What signs or symptoms did you have during this illness?

	1st Illness		2nd Illness		3rd Illness	
**Signs/Symptoms**	**Yes**	**No**	**Yes**	**No**	**Yes**	**No**
Fever						
Weakness/Lethargy						
Headache						
Body / muscle pain						
Joint pain						
Cough						
Abdominal Pain						
Nausea						
Vomiting						
Diarrhea						
Jaundice (yellowing of the skin)						
Bruising						
Petechiae (small dark purple or dark red dots that don't go away when you push down on them)						
Nose Bleeding						
Bleeding from gums						

Blood in vomitus						
Blood in stool						
Blood in urine						
Coughing blood						
Red Eyes						
Bleeding gums						
Other, please list:						

45. Did you seek any care for your symptoms?
    a. Yes
        i. Where? _____________
        ii. When? ______________
    b. No
        i. Why not? ______________________ (End questionnaire)
46. If you were hospitalized, how long were you in the hospital for? ___________
47. Did you receive any medications or treatments?
    a. No
    b. Yes
        i. What? ________________
        ii. Received medication or treatment from (choose one answer only):
            a. Primary healthcare
            b. District
            c. Regional
            d. Tbilisi ID hospital
            e. Any other clinic in Tbilisi:________________
            f. Local pharmacy
            g. Local healer
            h. Other____________________
